# Learning curve and initial outcomes of a novel percutaneously endoscopic-assisted total hip arthroplasty through mini bikini direct anterior approach: an observational cohort study

**DOI:** 10.1186/s13018-024-04925-3

**Published:** 2024-07-20

**Authors:** Guiguan Wang, Yiyang Xu, Guoyu Yu, Fenqi Luo, Long Chen, Yuan Lin, Jie Xu

**Affiliations:** 1 Shengli Clinical College of Fujian Medical University, No.134 East Street, Fuzhou, Fujian China; 2 Department of Orthopedic, Fujian Provincial Hospital, No.134 East Street, Fuzhou, Fujian China; 3 Fujian Provincial Clinical Medical Research Center for Spinal Nerve and Joint Diseases, No.134 East Street, Fuzhou, Fujian China; 4 Fuzhou University Affiliated Provincial Hospital, No.134 East Street, Fuzhou, Fujian China

**Keywords:** Toal hip arthroplasty, Direct anterior approach, Percutaneously, Endoscopic-assisted, Learning curve

## Abstract

**Objective:**

Although the direct anterior approach can reduce muscle damage and ensure accurate prosthesis placement, the steep learning curve and increased risk of complications associated with DAA necessitate careful consideration. Therefore, we describe a technique for a novel percutaneously endoscopic-assisted total hip arthroplasty through mini bikini direct anterior approach (mDAA) and report the learning curve and initial outcomes.

**Methods:**

The first 125 THA performed by a single surgeon between September 2020 and February 2022 using the anterior approach were included, comprising the initial 41 cases of bikini DAA (bDAA) and the subsequent 84 cases of mDAA. Outcome measures included perioperative outcomes and postoperative complications. The cumulative sum analysis (CUSUM) was used to determine the learning curve of anterior approach THA for each patient’s ORT. Multivariable analysis was performed to determine risk correlation.

**Results:**

A total of 125 anterior approach THA completed between 2020 and 2022 were identified. Among these, 41 were performed via bDAA and 84 via mDAA. No statistically significant differences were observed between the groups in terms of age, gender distribution, BMI or follow-up time. A significant reduction in ORT was noted, from 140 min for bDAA to 130 min for mDAA. Furthermore, there was a consistent decrease in LOI, LOS, and wound-healing problems. There was no statistically significant difference between groups with respect to Harris Hip Scores and other postoperative complications. The curve inflection points of the learning curve for the bDAA and mDAA group were located in the 22nd and 68th cases, respectively. The reduction of hemoglobin indicated a predicted increase in ORT.

**Conclusions:**

In this study, ORT, LOI, LOS, and wound-healing problems decreased overall in mDAA group. After mastering the bDAA technique, approximately 27 mDAA cases are needed to acquire proficiency in this technique. Hence, mDAA is a valuable alternative for those seeking smaller incisions, resolving wound healing problems, and aiming for enhanced recovery after surgery.

## Introduction

The direct anterior approach (DAA) for total hip arthroplasty (THA) is performed in a supine position and exploits an anatomical plane between the tensor fasciae latae and sartorius muscles [1]. In contrast to conventional lateral or posterior approaches, the anterior approach presents notable benefits, notably the avoidance of abductor muscle splitting, potentially mitigating the occurrence of gait disturbances, while concurrently diminishing the dislocation rates [2]. Despite advancements, the conventional direct anterior approach (cDAA) remains encumbered by several constraints. Primarily, scholarly discourse characterizes cDAA as possessing a steep learning curve [3]. Furthermore, literature suggests that cDAA, with its vertical incision perpendicular to the skin creases, may lead to increased skin tension, delayed wound healing and subsequent scar formation [4]. The paramount consideration lies in the substantial elevation of the risk of major surgical complications [5].

The bikini direct anterior approach (bDAA) employs a transverse incision aligned with Langer’s lines, enhancing patients' satisfaction with postoperative scars [6, 7]. Additionally, the incision location is closer to the femoral neck, facilitating easier femoral osteotomy and reducing the need for muscle dissection and traction. However, bDAA incisions are even shorter and constrained surgical visibility is noted. Consequently, reliance on offset instrumentation is common during the procedure, potentially exacerbating pressure on the skin edges and precipitating recurrent wound healing problems which leads to approximately 14.2% of patients still experience wound problems [7]. Therefore, we propose the utilization of modular instrumentation and introduce a novel percutaneously endoscopic-assisted total hip arthroplasty through mini bikini direct anterior approach (mDAA), which involves surgery through a mini-open bikini incision approximately 5–6 cm in length and a distal puncture site of 1 cm for acetabular reaming and cup impaction. Previous findings indicated that the mDAA exhibited favourable clinical efficacy, with a notable reduction in the length of incision while reducing wound problems in obese patients [8].

The primary objective of this study is to assess the learning curves of the same surgeon who learns bDAA and subsequently refines mDAA. The secondary objective is to investigate whether mDAA, in comparison to bDAA, can improve clinical outcomes and reduce surgical complications. We hypothesize that mDAA would decrease the learning curve while maintaining patient safety.

## Materials and methods

### Ethics approval

This study was designed as a retrospective study in line with the principles of the Declaration of Helsinki and approved by the Independent Ethics Committee. All participants provided informed consent for inclusion before their participation in the study. And this work has been reported in line with the STROCSS criteria [9].

### Patient selection

The initial 125 cases of bDAA and mDAA THA, conducted by the same surgeon and adhering to the inclusion and exclusion criteria, were retrospectively enrolled from September 2020 to February 2022.

Inclusion criteria included: (i) patients underwent primary THA via bDAA, or mDAA; and (ii) patients diagnosed with avascular necrosis of the femoral head, femoral neck fracture, congenital hip dysplasia (Crowe type 1, 2), osteoarthritis, rheumatoid arthritis, or ankylosing spondylitis.

Exclusion criteria included: (i) patients with other hip fractures other than the femoral neck; (ii) patients undergoing revision hip arthroplasty; (iii) severe congenital hip dysplasia (Crowe type 3, 4); (iv) combined with serious diseases affecting postoperative rehabilitation exercise, such as severe knee joint disease, spinal disease, etc.; and (v) incomplete follow-up data of patients.

Preoperative characteristics including age, gender, BMI, ASA classification, follow-up time and predicted blood volume were collected. The predicted blood volume for each patient was conducted using the method described by Nadler [10]. This method calculates blood volume based on the patient's height and weight.

### Surgical technique

All surgical procedures were performed by the same surgeon who had not previously encountered bDAA. The surgeon had prior extensive experience with completing posterior and lateral approaches.

### Anesthesia and position

The patients were positioned supine, with their pubic symphysis aligned with the fold of the operating table. A “cocktail mixture” (composed of 150 mg of ropivacaine, 1/2 vial of adrenaline 1:10,000, and diluted with physiological saline to 100 ml) of local infiltration analgesia was administered prior to making the skin incision.

### Approach and exposure

The mDAA approach has been elucidated in a previous study [8]. An incision measuring approximately 5 ~ 6 cm in length was made in the lateral groin crease. To minimize lateral cutaneous femoral nerve (LFCN) injury, the length of the incision should constitute two-thirds of the total length along the lateral aspect of the anterior superior iliac spine line. Proceed by making a horizontal cut through the skin and subcutaneous layer, followed by a vertical incision through the deep fascial tissue. Upon identifying the interval between the tensor fasciae latae and the sartorius muscle, access to the Hueter interval was established. The subcutaneous tissue on the medial side of the Hueter gap was separated and retracted medially to protect the LFCN. Through the Hueter gap, the lateral femoral circumflex artery was identified and dissected. The articular capsule was incised to form a flap. A Hohmann retractor was used to protect the LFCN. Employing a two-knife technique, the femoral neck was excised to ensure the femoral head could be removed separately through the minimally invasive incision.

Using a “finger-touch” technique, a percutaneously-assisted channel of approximately 1 cm was placed at a muscle interval about 10 cm distal to the primary incision. Selectively placed a commonly used disposable laparoscopic trocar at the percutaneously-assisted channel. Preparation of the acetabulum was performed under endoscopy. The acetabular reamer was placed through the main incision, and the handle was introduced through the percutaneous access. Once assembled, it was used to ream the acetabulum to the preoperatively planned depth. After placing the acetabular component through the main incision and adjusting it to the proper position, an impactor was placed through the percutaneously-assisted channel to impact the prosthesis until it was stabilized.

The operating table was adjusted to hyperextend the hip joint by approximately 30–40°. A lift-top tractor (Chinese National Patent, Patent Number: ZL201821970909.X) was utilized to elevate the proximal femur. Subsequently, the medullary cavity was reamed based on preoperative measurements. Following assessment of lower limb length, prosthesis stability, joint mobility, and impingement phenomenon, the femoral stem prosthesis and femoral head were inserted.

In the bDAA group, the incision length is approximately 6–8 cm, located 2 cm distal to the anterior superior iliac spine. Meanwhile, percutaneously-assisted channels are not used during the surgery.

### Perioperative care

All patients received the same standardized perioperative treatment regimen. Antibiotics and tranexamic acid were administered via intravenous infusion preoperatively. Patients promptly commenced a standardized multimodal analgesia regimen postoperatively. Early mobilization with weight-bearing exercises was encouraged on the day of the operation. Oral administration of rivaroxaban was routinely recommended for a duration of 5 weeks postoperatively.

### Outcomes

All patients were followed-up at 2 weeks, 6 weeks, 6 months and 12 months. The followed-up results were obtained from the prospectively established clinical records database. The primary study outcome was the operating room time (ORT), defined as the total duration in minutes from entering to exiting the operating room for it has been proved to be the most difficult outcome to improve. Secondary outcomes include perioperative outcomes and postoperative complications. Perioperative outcomes encompass the length of incision (LOI), reduction of hemoglobin in grams per deciliter, transfusion requirements, and length of stay (LOS). Postoperative complications include Harris Hip Scores at 12 months (HHS), wound-healing problems (wound ooze and delayed wound healing), venous thrombo embolism (VTE), LFCN dysesthesia (defined as numbness in the region innervated by LFCN), infection, revision, periprosthetic fracture, and dislocation.

### Statistical analysis

For normally distributed continuous variables, data were presented as means and standard deviations and intergroup differences were assessed for significance using Student t-test. Otherwise, the data were presented as median and quartiles, and were compared using the Mann–Whitney U test. χ2 tests were applied for categorical variables. The learning curve was analysed using the cumulative sum method (CUSUM). This involved employing cumulative sums to assess the ORT across a sequential series of operations, aimed at determining proficiency in surgical performance and if the learning curve was overcome. Fitting curves based on the increase in cases according to ORT, representing estimated ORT. Generalized linear modeling was used to model ORT of the mDAA group. Multivariable analysis was performed in a stepwise analysis and presented as odds ratios (ORs) with 95% confidence intervals, estimated probability and coefficients to ORT of the mDAA group. All statistical tests were 2-sided, and differences were considered significant when P was less than 0.05. Statistical analyses were performed using Python 3.12 and SPSS 22.0.

## Results

### Patient demographics

A total of 125 consecutive patients underwent bikini direct anterior approach, 41 in the bDAA group and 84 in the mDAA group. No significant differences were observed in age (median [IQR]: 59.00 [42.00, 68.00] *vs.* 57.50 [51.00, 68.75]), gender distribution (*p* = 0.532), BMI (median [IQR]: 23.43 [21.71, 24.99] *vs.* 23.38 [21.31, 26.66]), ASA classification (*p* = 0.249), predicted blood volume (median [IQR]: 3.98 [3.62, 4.72] *vs.* 3.99 [3.63, 4.42]), or follow-up time (median [IQR]: 12 [12, 12.5]*vs.* 12 [12, 12]) (Table 1).

**Table 1:** Cohort characteristics by approaches

Variables	Approach		*p* value
	bDAA (n = 41)	mDAA (n = 84)	
Preoperative characteristics			
Age (years)	59.00 [42.00, 68.00]	57.50 [51.00, 68.75]	0.455
Gender			0.532
Female (n)	21 (51.2%)	36 (42.9%)	
Male (n)	20 (48.8%)	48 (57.1%)	
BMI (kg/m^2^)	23.43 [21.71, 24.99]	23.38 [21.31, 26.66]	0.636
ASA classification			0.249
1 (n)	1	0	
2 (n)	14	39	
3 (n)	26	44	
4 (n)	0	1	
Predicted blood volume (L)	3.98 [3.62, 4.72]	3.99[3.63, 4.42]	0.935
Follow-up months (m)	12 [12, 12.5]	12 [12]	0.856
Perioperative parameters			
ORT (min)	140 [130, 170.5]	130 [120, 150]	< 0.05
LOI (cm)	6 [5, 6.5]	5 [5, 6]	< 0.05
Transfusion (n)	3 (7.3%)	5 (6.0%)	0.770
Reduction of hemoglobin (g/dL)	1.9 [1.35, 2.95]	2.25 [1.425, 3.275]	0.171
LOS (d)	4 [2, 5]	3 [2, 3]	< 0.05
Postoperative outcomes			
HHS	93.19 ± 4.32	93.63 ± 3.63	0.578
Wound-healing problems	8 (19.5%)	5 (7.1%)	< 0.05
Wound ooze (n)	6 (14.6%)	4 (4.7%)	0.203
Delayed wound healing (n)	2 (4.9%)	1 (1.2%)	0.521
VTE	2 (4.9%)	3 (3.6%)	0.726
LFCN dysesthesia	5 (12.1%)	7 (8.3%)	0.491
Infection	0	0	–
Revision	0	0	–
Periprosthetic fracture	1 (2.4%)	0	0.713
Dislocation	0	0	–

*bDAA* bikini DAA, *mDAA* percutaneously endoscopic-assisted total hip arthroplasty through mini bikini direct anterior approach, *BMI* body mass index, *ASA* American Society of Anesthesiologists, *ORT* operating room time, *LOI* length of incision, *LOS* length of stay, *HHS* Harris Hip Score, *VTE* venous thrombo embolism, *LFCN* lateral femoral cutaneous nerve, *SD* standard deviation, *IQR* interquartile range

Results from normal distribution are represented by mean ± SD

Results from non-normal distribution are represented by median [IQR]

Statistically significant values at *p* less than 0.05

### Perioperative parameters

Perioperative outcomes revealed significant improvements with mDAA, manifesting in reduced ORT (median [IQR]: 140 [130, 170.5] *vs.* 130 [120, 150] minutes, *p* < 0.05) and LOI (median [IQR]: 6 [5, 6.5] *vs.* 5 [5, 6] cm, *p* < 0.05). Additionally, mDAA exhibited shorter LOS (median [IQR]: 4 [2, 5] *vs.* 3 [2, 3] days, *p* < 0.05) (Table 1).

### Postoperative outcomes

Postoperatively, wound-healing complications were significantly lower in the mDAA group, including wound-healing problems (19.5% *vs.* 7.1%, *p* < 0.05) and wound ooze (14.6% *vs.* 4.7%, *p* = 0.203). Other postoperative outcomes such as HHS, VTE, LFCN dysesthesia, and incidence of infection, revision, periprosthetic fracture, and dislocation did not exhibit significant differences between the two groups (Table 1).

### Learning curve and multivariable analysis of ORT

**Fig. 1:**
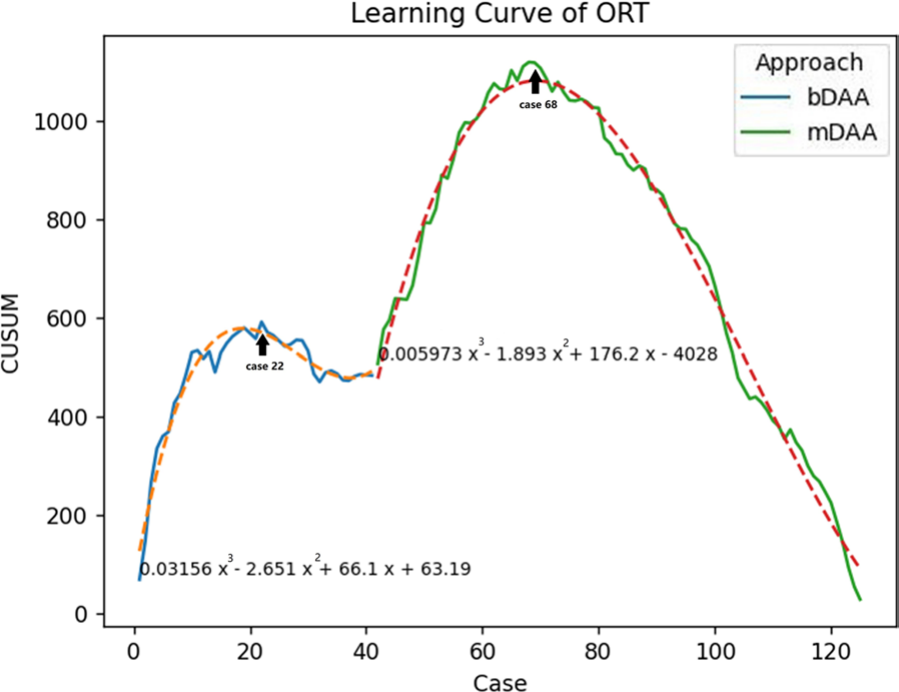
The CUSUM chat for ORT of bDAA and mDAA. Abbreviations: ORT: operating room time; bikini DAA: bDAA; percutaneously endoscopic-assisted total hip arthroplasty through mini bikini direct anterior approach: mDAA. →: the inflection point

**Fig. 2:**
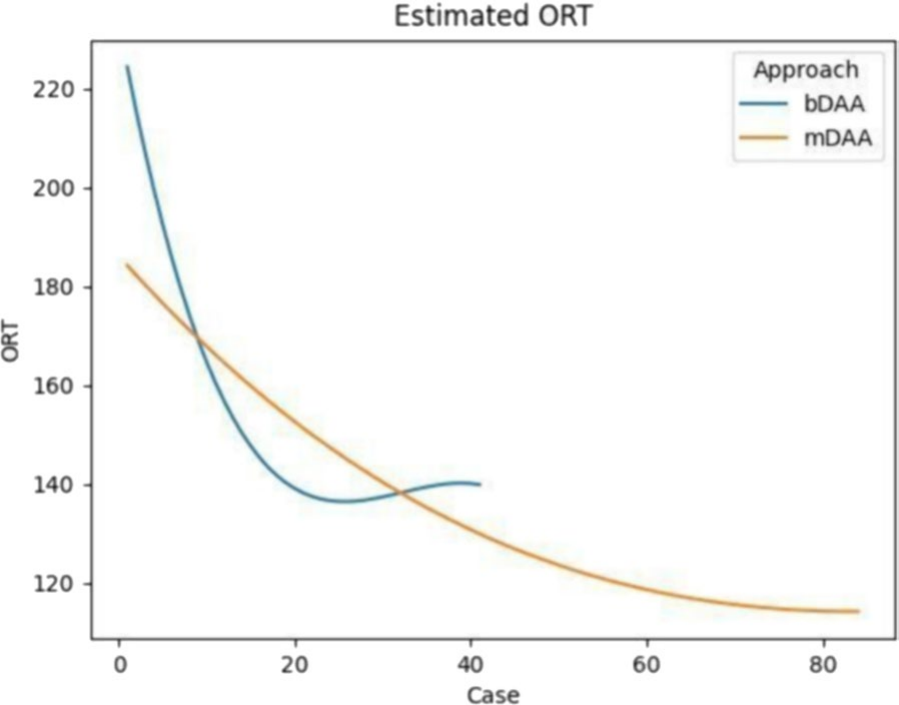
The estimated ORT of bDAA and mDAA. Abbreviations: ORT: operating room time; bikini DAA: bDAA; percutaneously endoscopic-assisted total hip arthroplasty through mini bikini direct anterior approach: mDAA

The CUSUM learning curve was effectively characterized by cubic equation models, wherein: CUSUM (min) = 0.03x^3 ^− 2.65x^2^ + 66.61x + 63.19 for bDAA, and 0.005x^3 ^− 1.189x^2^ + 176.2x − 4028 for mDAA, with the latter initiating from 42 cases (Fig. 1). The curve inflection points of the learning curve are located in the 22nd and 68th cases, dividing the learning curve into the learning phase and the experience phase. Crossing the inflection point of the learning curve implies that ORT consistently stabilizes below the average level, indicating that the surgeon has mastered the surgical technique. Figure 2 depicts the fitted curves of ORT for bDAA and mDAA groups respectively. The results reveal that the mDAA group exhibits a lower initial ORT and a smoother curve fit. Only the reduction of hemoglobin was identified as a factor contributing to increased ORT. Age, case, BMI, and LOI were found to have no significant influence on ORT (Table 2).

**Table 2:** Multivariable analysis of factors associated with ORT of the mDAA group

Factors	Coefficient	*p* value
Age (years)	0.039	0.692
Case (n)	− 0.074	0.447
BMI (kg/m^2^)	− 0.070	0.475
LOI (cm)	− 0.070	0.477
Reduction of hemoglobin (g/dL)	0.478	< 0.01

*ORT* operating room time, *mDAA* percutaneously endoscopic-assisted total hip arthroplasty through mini bikini direct anterior approach, *BMI* body mass index, *LOI* length of incision

Positive coefficients indicate a predicted increase in ORT, whereas negative coefficients suggest a predicted decrease in ORT

Statistically significant values at *p* less than 0.05

## Discussion

The principal finding of this study was that the novel percutaneously endoscopic-assisted total hip arthroplasty through mini bikini direct anterior approach is a safe and reliable surgical approach, with a relatively smooth learning curve while reducing complications within 1 year compared to those who had bikini DAA THA.

The selection of surgical approach in THA remains a topic of debate. The anterior approach is associated with quicker postoperative recovery due to reduced muscle trauma, potentially leading to decreased pain and improved functional outcomes in early stage [2]. However, the risk of major surgical complications associated with DAA is also significant and cannot be overlooked [5]. To address perioperative femoral fractures and wound-healing problems, bDAA was proposed [11]. However, due to the limited incision size, which restricts the surgical field, the use of offset instruments is often necessary in bDAA. Additionally, the increased tension exerted by surgical instruments on the skin edges may lead to postoperative wound ooze and other wound-healing problems [7]. Percutaneously assisted total hip arthroplasty was first attempted by Riley et al. in 2004 [12]. Through their specially designed instruments, it is possible to accomplish THA via the posterior approach, preserving the soft tissues and allowing full visualization and access without compromising component positioning [13, 14]. The innovation of the mDAA lies in its introduction of the learning curve of percutaneously-assisted concepts into the anterior approach for the first time to our knowledge. This enables surgeons to significantly minimize incision length under supine-positioned DAA while restoring visualization and reducing the occurrence of wound healing problems based on the current findings. Wang's study reported a wound healing problem rate of 14.2%, similar to our bDAA group [7]. However, in the subsequent mDAA group, there was a significant reduction in wound healing problems. This may be related to the use of modular instruments during surgery and a reduction in skin edge tension.

The learning curve associated with DAA is frequently characterized as steep and arduous [15]. Changes in surgical positioning, unfamiliarity with anatomical structures, restricted surgical visibility and limited access for the introduction of components are all factors that can greatly impact the learning curve. According to existing evidence, it generally takes a surgeon 40–100 cases to master the DAA technique and reach an acceptable and steady state [15–18]. However, in this study, the surgeon reached a turning point in the learning curve around the 22nd case. This may be due to the surgeon's prior experience with cDAA and subsequently becoming more familiar with bDAA for supine position THA [19]. Whereas, during the learning curve, there is a substantial likelihood of increased perioperative complications. In our study, the bDAA group experienced a major surgical complication early in the learning curve, which may be attributed to insufficient surgical experience resulting from the change in surgical approach. Hence, Lawrie proposed total hip arthroplasty via the direct anterior approach in the lateral decubitus position and found that this approach showed no detectable learning curve effect [20]. However, compared to the DAA in lateral decubitus position, mDAA maintains the supine position, simplifying preoperative preparation and improving anesthesia management. Simultaneously, the supine position facilitates intraoperative fluoroscopy and optimizing component positioning [21]. In the mDAA approach we describe, we found a relatively smooth learning curve, with the inflection point occurring at 68 cases (the 27th case after switching to mDAA), and there were no occurrences of major surgical complications within the mDAA group. Currently, there is a lack of research on the learning curve of bDAA. Moreover, in comparison to cDAA, bDAA have more limited incisions and narrower fields of view. Under these circumstances, we consider the learning curve achieved with mDAA to be acceptable.

A stepwise analysis approach was employed in the multivariable analysis to systematically exclude confounding variables including age, case number (indicating surgical experience), BMI, and LOI from ORT. By this approach, it was determined that the reduction of hemoglobin remained significantly associated with the increasement of ORT. This finding indicated that despite the standardized perioperative management, intraoperative blood loss, as reflected by the reduction in hemoglobin, independently contributes to longer surgical times. The reduction of hemoglobin reflected the complexity and invasiveness of the surgery, which directly impacts the duration of the procedure. Thus, while the reduction of hemoglobin is not an independent variable in the strictest sense, its significant association with ORT underscores its role as a critical factor in surgical planning and execution. Additionally, the lack of correlation between BMI and ORT suggests promising prospects for the application of mDAA in obese patients.

The addition of an extra percutaneously-assisted channel in the procedure does not result in a prolonged operating room time. The percutaneously-assisted channel provides an additional operating pathway for surgical instruments, allowing the light source to access the surgical field and enabling the possibility of using endoscopic assistance when necessary. Moreover, through this channel, the use of modular instruments allows surgeons to better perform acetabular reaming and cup impaction under direct visualization [12]. Hence, we found that in the mDAA group, both the ORT and LOI were significantly shorter than those in the bDAA group.

Despite the numerous benefits of mDAA, the shortened incision still fails to reduce the occurrence rate of LFCN dysesthesia despite a decreasing trend. This trend may be attributed to the reduction in incision size and the decreased pressure on surrounding soft tissues due to the use of modular instruments. Additionally, the gradual recovery of LFCN dysesthesia during postoperative follow-up suggests that this reversible damage is likely not caused by anatomical injury [22, 23]. Accordingly, whether mDAA can reduce LFCN dysesthesia still requires a larger sample size for validation. Therefore, mDAA offers the advantages shared by bDAA while minimizing incision size and reducing wound healing problems.

The study has several limitations, with the main one being that the learning curve was based on the experience of a single expert surgeon who transitioned from mastering bDAA to exploring mDAA. Therefore, this learning curve may not be reproducible in other surgeons. Although for surgeons who have mastered DAA, this curve may be smoother. Another limitation lies in the selection bias and missing data inherent in retrospective studies. Furthermore, we only conducted follow-up assessments up to 1 year postoperatively. Long-term follow-up is needed, along with comparative studies against classic surgical approaches including the posterior or the lateral approach, to better understand the advantages of mDAA.

## Conclusion

mDAA was associated with a smooth learning curve, enabling the surgeon to achieve maximum reduction in incision size without compromising patient safety. Conservatively, after acquiring proficiency in the bDAA technique, approximately 27 cases are needed to achieve full mastery of mDAA. Based on the present findings, we suggest that surgeons seeking to minimize incision size and patients prone to postoperative wound healing issues consider transitioning to mDAA.

## Data Availability

The original contributions presented in the study are included in the article. Further inquiries can be directed to the corresponding authors.
